# Left ventricular global function index assessed by cardiac magnetic resonance imaging for the prediction of cardiovascular events in ST-elevation myocardial infarction

**DOI:** 10.1186/1532-429X-17-S1-O34

**Published:** 2015-02-03

**Authors:** Ingo Eitel, Janine Pöss, Alexander Jobs, Charlotte Eitel, Suzanne de Waha, Jörg Barkhausen, Steffen Desch, Holger Thiele

**Affiliations:** 1 Cardiology, Angiology, Intensiv CAre Medicine, Heart Center Lübeck, Lübeck, Germany; 2 Cardiology, Heart Center Bad Segeberg, Bad Segeberg, Germany; 3 Radiology, University Lübeck, Lübeck, Germany

## Background

The left ventricular performance index (LVGFI) as a comprehensive marker of cardiac performance integrates LV structure with global function within one index. In a prospective cohort study of healthy individuals the LVGFI demonstrated a superior prognostic value as compared to LV ejection fraction (LVEF). In patients after ST-segment elevation myocardial infarction (STEMI), however, the role of the LVGFI is unknown. Aim of this study was to investigate the relationship between the LVGFI and infarct characteristics as well as prognosis in a large multicenter STEMI population.

## Methods

In total 795 STEMI patients reperfused by primary angioplasty (<12 h after symptom onset) underwent cardiac magnetic resonance (CMR) imaging at 8 centers. CMR was completed within one week after infarction using a standardized protocol including LV dimensions, mass and function for calculation of the LVGFI. The primary clinical endpoint of the study was the occurrence of major adverse cardiac events (MACE).

## Results

The median LVGFI was 31.2% (interquartile range 25.7 to 36.6). Patients with LVGFI<median had significantly larger infarcts, less myocardial salvage, a larger extent of microvascular obstruction, higher incidence of intramyocardial hemorrhage and more pronounced LV dysfunction (p<0.001 for all). MACE and mortality rates were significantly higher in the LVGFI <median group (p<0.001 and p=0.003, respectively). The LVGFI had an incremental prognostic value in addition to LVEF for prediction of all-cause mortality.

## Conclusions

The LVGFI strongly correlates with markers of severe myocardial and microvascular damage in patients with STEMI, offering prognostic information beyond traditional cardiac risk factors including the LVEF.

## Funding

None.

